# Biochemical and structural characterization of a thermostable *β*-glucosidase from *Halothermothrix orenii* for galacto-oligosaccharide synthesis

**DOI:** 10.1007/s00253-014-6015-x

**Published:** 2014-08-31

**Authors:** Noor Hassan, Thu-Ha Nguyen, Montira Intanon, Lokesh D. Kori, Bharat K. C. Patel, Dietmar Haltrich, Christina Divne, Tien Chye Tan

**Affiliations:** 1 KTH Royal Institute of Technology, School of Biotechnology, Albanova University Center, Roslagstullsbacken 21, S-10691 Stockholm, Sweden; 2 Food Biotechnology Laboratory, BOKU University of Natural Resources and Life Sciences, A-1190 Vienna, Austria; 3 Microbial Gene Research and Resources Facility, Griffith University, School of Biomolecular and Physical Sciences, Brisbane, QLD 4111 Australia; 4 Biochemistry and Molecular Biology, Baylor College of Medicine, Houston, TX 77030 USA; 5 Department of Medical Biochemistry and Biophysics, Scheelelaboratoriet, Karolinska Institute, Scheeles väg 2, S-17177 Stockholm, Sweden

**Keywords:** *β*-glucosidase, *β*-galactosidase, Halothermophile, *Halothermothrix*, Lactose conversion, Galacto-oligosaccharides, Biochemical characterization, Structural analysis

## Abstract

Lactose is a major disaccharide by-product from the dairy industries, and production of whey alone amounts to about 200 million tons globally each year. Thus, it is of particular interest to identify improved enzymatic processes for lactose utilization. Microbial *β*-glucosidases (BGL) with significant *β*-galactosidase (BGAL) activity can be used to convert lactose to glucose (Glc) and galactose (Gal), and most retaining BGLs also synthesize more complex sugars from the monosaccharides by transglycosylation, such as galacto-oligosaccharides (GOS), which are prebiotic compounds that stimulate growth of beneficial gut bacteria. In this work, a BGL from the thermophilic and halophilic bacterium *Halothermothrix orenii*, *Ho*BGLA, was characterized biochemically and structurally. It is an unspecific β-glucosidase with mixed activities for different substrates and prominent activity with various galactosidases such as lactose. We show that *Ho*BGLA is an attractive candidate for industrial lactose conversion based on its high activity and stability within a broad pH range (4.5–7.5), with maximal *β*-galactosidase activity at pH 6.0. The temperature optimum is in the range of 65–70 °C, and *Ho*BGLA also shows excellent thermostability at this temperature range. The main GOS products from *Ho*BGLA transgalactosylation are β-d-Gal*p*-(1→6)-d-Lac (6GALA) and β-d-Gal*p*-(1→3)-d-Lac (3GALA), indicating that d-lactose is a better galactosyl acceptor than either of the monosaccharides. To evaluate ligand binding and guide GOS modeling, crystal structures of *Ho*BGLA were determined in complex with thiocellobiose, 2-deoxy-2-fluoro-d-glucose and glucose. The two major GOS products, 3GALA and 6GALA, were modeled in the substrate-binding cleft of wild-type *Ho*BGLA and shown to be favorably accommodated.

**Electronic supplementary material:**

The online version of this article (doi:10.1007/s00253-014-6015-x) contains supplementary material, which is available to authorized users.

## Introduction

Lactose generated as by-product from liquid whey alone amounts to an impressive 150–200 million tons per year (Smithers 2008). Thus, lactose constitutes a vast carbohydrate resource for industrial enzymatic processes towards value-added products with the aim to promote sustainable development. One important application of lactose conversion is the production of compounds such as galacto-oligosaccharides (GOS). GOS have been recognized as prebiotic compounds that stimulate growth of certain members of the gut microbiota associated with beneficial effects. Production of GOS from lactose can be achieved by different approaches: (i) transglycosylation activity by glycoside hydrolases (GHs), which is the method currently employed by industry for production of GOS from lactose, with the yield of the reaction depending on the relative ratio of the transglycosylation versus hydrolysis reaction; (ii) acid hydrolysis of lactose, which produces a complex mixture of disaccharides and trisaccharides with a variety of linkages and anomeric configurations; however, this latter approach results in glycoside preparations that are non-applicable to the food industry since they do not meet the EC food regulations (De Roode et al. 2003); (iii) the use of glycosyltransferases to synthesize the sugar compounds of interest. Glycosyltransferases use activated sugar nucleotide donors where the sugar is transferred to a specific acceptor. Although the reactions are highly stereoselective and regioselective to give defined products compared with the use of glycoside hydrolases and chemical acid hydrolysis, the method is costly since the cost of enzyme production is high and the sugar nucleotide donors are expensive.

Based on the advantages and disadvantages of the different approaches mentioned above, the use of GHs is preferred from the perspective of purpose and cost, at least when GOS production for the food industry is considered. However, the yield of GOS formed by GHs through transglycosylation is an issue and needs to be addressed in each individual case, i.e., depending on the enzyme(s) and reaction conditions used. There are strategies for improving GOS yields, such as selecting an enzyme with high inherent transglycosylation activity. Although examples of engineered GH mutants with increased transglycosylation activity have been reported (Jørgensen et al. 2001; Placier et al. 2009; Wu et al. 2013), the mechanism behind the altered reaction patterns is not understood. Other parameters that affect GOS yields include reaction temperature and lactose concentration. Due to the increased solubility of lactose at higher temperatures and the decrease in water available to act as acceptor, the GOS yield typically increases with increasing temperature (Vera et al. 2012). High temperature is also desirable to limit microbial contamination of the substrate solutions (Urrutia et al. 2013). As found by many other authors, the lactose concentration has a significant impact on the final GOS yield (Splechtna et al. 2006).

Restrictions on the temperatures that can be used will ultimately depend on the stability and activity profile of the enzyme catalyst, as well as the degree of side reactions in the reaction mixture, such as the Maillard reaction, i.e., glycosylation of mainly protein lysine side chains by reducing sugars. Bruins and coworkers reported that at temperatures above 80 °C, enzyme inactivation is doubled in the presence, as opposed to absence, of sugar (Bruins et al. 2003). Although this may not be an issue in batch-reaction mode, enzyme inactivation is likely to be more pronounced in a continuous system that operates over an extended time scale.

Based on the above considerations, enzymes that evolved naturally to tolerate high temperatures and concentrations of reaction substrates and products are of particular interest for industrial GOS production. Retaining BGALs used in the dairy industry are typically enzymes of fungal origin belonging to the GH2 family of glycoside hydrolases (http://www.cazy.org; Cantarel et al. 2009). As an alternative to BGALs, microbial *β*-glucosidases (BGLs; EC 3.2.1.21) can be used for the purpose of lactose conversion and GOS synthesis, as well as other biotechnological applications (Bhatia et al. 2002; Park et al. 2005). Unlike the BGALs of the GH2 family, most BGLs belonging to the GH1 family of glycoside hydrolases (http://www.cazy.org; Cantarel et al. 2009) are monomeric, small (50 kDa), and stable (α/β)_8_-barrel scaffolds where the functional catalytic property is built on a single polypeptide chain, thus making protein production easier and less costly. Moreover, GH1 BGLs typically display high β-galactosidase (BGAL) and transglycosylation activities. Another advantage of microbial BGLs in a biotechnological context is the broad specificity towards galactosidases, fucosidases, and xylosidases and the cleavage of *β*(1 → 1), *β*(1 → 2), *β*(1 → 4), and *β*(1 → 6) glycosidic bonds.

In the search of enzymes for lactose conversion and GOS production, it is of particular interest to screen genomes of thermophilic and hyperthermophilic bacteria for new enzyme candidates. *Halothermothrix orenii* is a heterotrophic, halophilic, thermophilic, and obligate anaerobic bacterium. The bacterium was originally isolated from a Tunisian hypersaline lake (Cayol et al. 1994), a habitat which is subjected to seasonal changes in temperatures and salinity. Indeed, bioinformatics analysis of the *H. orenii* genome revealed a full inventory of genes encoding GHs (Mijts and Patel 2001; Mavromatis et al. 2009), of which one gene coding for a GH1 *β*-d-glucosidase showed up as particularly interesting. The gene product was named *Ho*BGLA, and a preliminary structural X-ray characterization was performed at 3.0 Å resolution (Kori et al. 2011). However, the crystals of this enzyme variant were of poor quality and not useful for further crystal-structure analysis of ligand complexes and rational enzyme design.

Here, we report the biochemical characterization and high-resolution structure analysis of wild-type *Ho*BGLA. We address specifically the enzyme’s catalytic performance for use in lactose conversion and GOS production, and show that the enzyme displays very promising characteristics for this application. The biochemical results are discussed with reference to the structural framework of *Ho*BGLA based on the new high-resolution crystal structures of wild-type *Ho*BGLA expressed with a cleavable hexahistidine tag. Additionally, the crystal structures of three complexes of wild-type *Ho*BGLA are reported: a covalent *Ho*BGLA nucleophile (Glu354) complex with 2-deoxy-2-fluoro-d-glucose at 2.0 Å resolution; *Ho*BGLA in complex with thiocellobiose at 1.85 Å resolution; and a *Ho*BGLA complex with d-glucose (Glc) at 1.80 Å resolution. To confirm the identity of the catalytic residues, activity data using catalytically compromised active-site mutants are included in our analysis, i.e., variants where the acid/base catalyst (Glu166) and the nucleophile (Glu354), as well as a crucial substrate-binding side chain (Glu408) have been replaced by isosteric glutamine side chains.

## Materials and methods

### Cloning, expression, and purification of wild-type HoBGLA

The cloning of the *H. orenii bglA* gene (1.35 kbp) coding for 451 amino acids (UniProtKB B8CYA8) into the expression vector pET22b(+) carrying a non-cleavable C-terminal hexahistidine tag has been reported previously (Kori et al. 2011). Since the expressed protein from this gene construct resulted in poorly diffracting protein crystals (maximum 3.0 Å resolution), the same *bglA* gene was also cloned into an alternative *Escherichia coli* expression vector. The *bglA* gene was amplified by standard PCR and cloned into the pNIC28-Bsa4 vector under the control of T7 promoter (Savitsky et al. 2010) using ligation-independent cloning (Doyle 2005). The vector adds a cleavable hexahistidine tag and the Tobacco Etch virus (TEV) protease cleavage site at the N-terminus of the expressed protein with the sequence ^−23^MHHHHHHSSGVDLGTENLYFQSM^−1^, which allows for the tag to be removed proteolytically using TEV protease. The recombinant plasmid expressing His_6_-TEV-*bglA* was initially transformed into *E. coli* Mach1™ (Invitrogen) and grown on Luria Bertani (LB) agar plates supplemented with 5 % sucrose and 50 μg/mL kanamycin for the selection of recombinant plasmids with cleaved SacB (levansucrase).

The recombinant plasmid was isolated from *E. coli* Mach1™ cells using plasmid preparation QIAprep® Spin Miniprep Kit (Qiagen), followed by transformation into the *E. coli* expression strain BL21(DE3). Transformed BL21(DE3) cells were grown in 0.6 L Terrific Broth (TB) medium supplemented with 50 μg/mL kanamycin and 60 mL glycerol (per 600 mL), inoculated with 7 mL overnight seed culture of transformed BL21 (DE3), and allowed to grow at 37 °C with constant shaking at 200 rpm. At an optical density (OD) at 600 nm of 0.7, *bglA* expression was induced with 0.2 mM *β*-d-1-thiogalactopyranoside (IPTG) and the culture was left to grow at 18 °C for 16 to 18 h. Cells were harvested by centrifugation at 4 °C (8983 *rcf*) using an Avanti J-20 XP centrifuge (Beckman) with rotor JLA 8.1000 for 15 min. The bacterial cell pellet was resuspended in three volumes of lysis buffer [20 mM 4-(2-hydroxyethyl)-1-piperazine ethanesulfonic acid (HEPES) pH 7.0, 150 mM NaCl]. The sample was homogenized using an AVESTIN Emulsiflex-C3 system, and the lysate was collected in a beaker on ice. The lysate was centrifuged at 4 °C, (39191 *rcf*) using an Avanti J-20 XP centrifuge (Beckman) with rotor JA 25.50 for 30 min to pellet the cell debris.

The gene product resulting from expression of pNIC28-Bsa4-*bglA* is hereafter denoted *Ho*BGLA, and the gene product expressed from the pET22b(+) vector as *Ho*BGLA_*PET*_. A 2-mL Ni^2+^-charged immobilized metal affinity chromatography (IMAC) Ni-NTA agarose resin (Invitrogen) was washed and equilibrated with lysis buffer. Clear lysate containing *Ho*BGLA was loaded onto the column, followed by a washing step with five column volumes (CVs) of wash buffer (20 mM HEPES pH 7.0, 150 mM NaCl, and 20 mM imidzaole). *Ho*BGLA was eluted with elution buffer (20 mM HEPES pH 7.0, 150 mM NaCl, and 350 mM imidazole). To cleave off the hexahistidine tag and remove the imidazole, the protein sample was treated with TEV protease at 1:50 ratio, placed in a dialysis bag with molecular weight cut off (MWCO) 12–14 kDa, which was incubated in a beaker containing dialysis buffer (20 mM HEPES pH 7.0 and 150 mM NaCl) at 4 °C overnight. Following TEV protease treatment, *Ho*BGLA was subjected to a second round of Ni^2+^-IMAC purification. The flow-through containing the TEV-treated protein was collected.

The protein sample was concentrated to 35 mg/mL using a Vivaspin® centrifugal concentrator (MWCO 10 kDa). To remove any Ni^2+^ contamination from the previous IMAC steps, EDTA was added to a final concentration of 10 mM before the sample was further purified by size-exclusion chromatography using a HiLoad^TM^ 16/60 Superdex^TM^ 200 prep grade column (GE Healthcare Life Sciences) equilibrated with 20 mM HEPES (pH 7.0) and 150 mM NaCl. Protein-containing fractions (1 mL) were collected. Suitably pooled fractions were concentrated to 50 mg/mL and used for subsequent crystallization experiments. The purity of *Ho*BGLA was assessed by SDS-PAGE.

### Production of catalytically impaired mutants of HoBGLA

To generate *Ho*BGLA mutants E166Q, E354Q, and E408Q, the *Ho*BGLA gene from the vector pNIC28-Bsa4-*bglA* was used as template and subjected to site-directed mutagenesis using PCR with the primers *Ho*BGLA E166Q_fwd (5′-TGGACCTCTGGGTTACCCATAATCAGCCCTGGGTAGTT-3′) and *Ho*BGLA E166Q_rev (5′-AACTACCCAGGGCTGATTATGGGTAACCCAGAGGTCCA-3′), *Ho*BGLA E408Q_fwd (5′-GGTTATTATGTGTGGTCATTGATGGATAATTTTCAGTGGGCC TATGGCTATAG-3′), and *Ho*BGLA E408Q_rev (5′-CTATAGCCATAGGCCCACTGAAAATTATCCA TCAATGACCACACATAATAACC-3′), as well as *Ho*BGLA E354Q_fwd (5′-CCGATAAGCCCCTTTACATAACACAGAACGGGGCAGCTTTT-3′) and *Ho*BGLA E354Q_rev (5′-AAAAGCTGCCCCGTTCTGTGTTATGTAAAGGGGCTTATCGG-3′), respectively. These primers were designed using the QuickChange® Primer Design Program from Agilent Technology.

The PCR reaction contained 0.5 U Pfu DNA polymerase (Fermentas, Germany), 70 ng of plasmid DNA, 0.4 μL primer pair (45 ng/μL), 10 μM of dNTP mix, 1 μL DMSO, 24 μL H_2_O, and 2.5 μL Pfu Ultra II Fusion buffer in a total volume of 25 μL. The following conditions were used for mutagenic PCRs: 30 cycles of 95 °C, 3 min; 94 °C, 30 s; 65 °C, 8 min; and with a final incubation at 65 °C for 10 min. Following PCR, the methylated template-DNA was degraded by digestion with 1 μL *Dpn*I (10 U) at 37 °C, 2 h.

The *Dpn*I-digested PCR products were initially transformed into the *E. coli* cloning strain Mach1™ (Invitrogen) grown on Luria Bertani (LB) agar plates supplemented with 50 μg/mL kanamycin. Recombinant plasmids from Mach1 cells were isolated using the QIAprep® Spin Miniprep Kit (Qiagen), followed by plasmid transformation into the *E. coli* expression strain BL21 (DE3). The *Ho*BGLA mutants E166Q, E354Q, and E408Q were expressed as for wild-type *Ho*BGLA.

### *β*-galactosidase and *β*-glucosidase activity assays with chromogenic substrates

When chromogenic *o*NPGal (*o*-nitrophenyl-β-d-galactopyranoside) or *p*NPGal (*p*-nitrophenyl-β-d-galactopyranoside) were used as substrates for *Ho*BGLA, the determination of *β*-galactosidase activity was carried out at 30 °C with 22 mM *o*NPGal or *p*NPGal solutions in 20 mM HEPES buffer containing 150 mM NaCl (pH 7.0). The reaction was initiated by adding 20 μL of enzyme solution to 480 μL of the substrate solution, and then incubated for 10 min using an Eppendorf thermomixer compact (Hamburg, Germany). Agitation was at 600 rpm. The reaction was stopped by adding 750 μL of 0.4 M Na_2_CO_3_. The release of *o*-nitrophenol (*o*NP) or *p*-nitrophenol (*p*NP) was measured by determining the absorbance at 420 nm. One unit of *o*NPGal or *p*NPGal activity was defined as the amount of enzyme releasing 1 μmol of *o*NP or *p*NP, respectively, per minute under the described conditions.

The *β*-glucosidase activity of *Ho*BGLA was measured using *o*NPGlc (*o*-nitrophenyl-*β*-d-glucopyranoside) or *p*NPGlc (*p*-nitrophenyl-*β*-d-glucopyranoside) as the substrates, in principle as described above for the *β*-galactosidase assay. One unit of *o*NPGlc or *p*NPGlc activity was defined as the amount of enzyme releasing 1 μmol of *o*NP or *p*NP, respectively, per minute under similar conditions as described for determination of *β*-galactosidase activity.

### Activity assays for wild-type and mutant HoBGLA with cellobiose and lactose as substrates

For characterization of the hydrolytic activity of *Ho*BGLA using cellobiose and lactose, the glucose oxidase (GOD) and horseradish peroxidase (POD) assays were used as described by Kunst and coworkers (Kunst et al. 1988). The assay solutions were prepared by adding GOD and POD to final concentrations of 2.41 and 1.45 U/mL, respectively, to 200 mL solution of 4 mM KH_2_PO_4_, 6.4 mM 4-aminoantipyrine, and 11 mM phenol pH 7.0.

When lactose or cellobiose was used as substrate, 20 μL enzyme solutions were added to 480 μL of substrate solution in 20 mM Bis-Tris buffer pH 7. The reaction mixtures were incubated at 50 °C using an Eppendorf heat block. After 5 min, the reaction was stopped by heating the reaction mixture at 99 °C for 3 min and the sample was centrifuged at 13,000 rpm for 1 min to pellet the protein precipitate. The sample was allowed to cool at room temperature, and the release of d-glucose was assessed colorimetrically by adding 60 μL of reaction mixture to 600 μL of the GOD/POD assay solution. The assay mixture (660 μL) was incubated in the dark at room temperature for 40 min, and the absorbance at 546 nm was measured. The amount of glucose produced was calculated from a glucose standard curve obtained by adding 60 μL (0.28–3.89 mM) of standard glucose solutions to 600 μL assay solution and incubated at room temperature in the dark for 40 min. One unit of lactase activity was defined as the amount of enzyme releasing 1 μmol of d-glucose per minute under the given conditions. One unit of cellobiose activity was defined as the amount of enzyme releasing 2 μmol of d-glucose per minute under similar conditions as described for determination of *β*-galactosidase activity using lactose as the substrate.

### Kinetic measurements

All steady-state kinetic measurements were performed at 65 °C using *o*NPGal, *p*NPGal, *o*NPGlc, *p*NPGlc, lactose, and cellobiose as substrates in 20 mM HEPES buffer containing 150 mM NaCl (pH 7.0) with the concentrations ranging from 0.5 to 20 mM for *o*NPGal and *p*NPGal, 0.1 to 15 mM for *o*NPGlc and *p*NPGlc, 10 to 700 mM for lactose, and 1 to 350 mM for cellobiose, respectively. The kinetic parameters were calculated by nonlinear regression, and the observed data were fit to the Henri–Michaelis–Menten equation (SigmaPlot, SPSS Inc., Illinois, USA).

### Temperature and pH profiles of the β-galactosidase activity of HoBGLA

The pH dependence of *Ho*BGLA activity was evaluated by the standard *β*-galactosidase assay with 22 mM *o*NPGal in the pH range from 4 to 10 using Briton–Robinson buffer (20 mM acetic acid, 20 mM phosphoric acid, and 20 mM boric acid titrated with 1 M NaOH to the desired pH). The temperature optima for the hydrolysis activity of *Ho*BGLA with both substrates lactose and *o*NPGal were determined at 30–85 °C. The thermostability was evaluated by incubating the pure enzyme in 20 mM HEPES and 150 mM NaCl (pH 7.0) at 65 and 70 °C. The residual activities were measured regularly with *o*NPGal as substrate. When lactose was used as substrate, the assay was carried out as previously described (Nguyen et al. 2007) with some modifications. The reaction was done in 20 mM HEPES buffer with 150 mM NaCl (pH 7.0) for 10 min at 30 °C, after which the reaction was stopped. The release of d-glucose was determined using a d-glucose assay kit (Megazyme). One unit of lactase activity was defined as the amount of enzyme releasing 1 μmol of d-glucose per minute under the given conditions.

### Transgalactosylation of lactose and analysis of galacto-oligosaccharides

Lactose solutions (200, 300, and 350 g/L) were prepared in 20 mM HEPES and 150 mM NaCl (pH 7.0) containing 1 mM MgCl_2_. Transgalactosylation reactions were performed on a 2-mL scale at 70 °C using 300 rpm agitation and 12 U_oNPGal_/mL final concentration of a homogenous preparation of *Ho*BGLA. Samples were withdrawn at specific time intervals and immediately transferred to 99 °C for 5 min to inactivate the enzyme. Samples were stored at −18 °C for subsequent analysis.

The GOS mixtures were analyzed by thin layer chromatography (TLC) and high-performance anion exchange chromatography with pulsed amperometric detection (HPAEC-PAD). TLC was carried out using high-performance TLC silica plates (HPTLC Lichrospher silica gel 60 F_254_S, Merck). An appropriately diluted sample containing ~20 g/L sugar was applied to the plate (1.0 μL) and eluted twice in ascending mode with an *n*-butanol/*n*-propanol/ethanol/water mixture (2:3:3:2). Thymol reagent was used for detection.

HPAEC−PAD analysis was carried out on a Dionex DX-500 system consisting of a GP50 gradient pump, an ED 40 electrochemical detector with a gold working electrode and an Ag/AgCl reference electrode, and Chromeleon version 6.5 (Dionex Corp., Sunnyvale, CA). All eluents were degassed by flushing with helium for 30 min. Separations were performed at room temperature on a CarboPac PA-1 column (4 × 250 mm) connected to a CarboPac PA-1 guard column (Dionex). Separation of d-glucose, d-galactose (Gal), lactose, and allolactose was carried out with an isocratic run (45 min) with 15 mM NaOH at 1.0 mL/min, followed by 25 min elution with 100 mM NaOH. For separation of other GOS, eluent A (100 mM NaOH) and B (100 mM NaOH and 150 mM NaAc) were mixed to form the following gradient: 98 % A from 0 to 10 min, 98 to 52 % A from 10 to 40 min, and then 52 % A for another 5 min. The column was washed with 20 % B for 10 min and re-equilibrated for 15 min with the starting conditions of the employed gradient. Individual GOS components were identified by comparison to authentic standard sugars.

### Crystal-structure analysis of HoBGLA ligand complexes

A preliminary X-ray crystallographic analysis at low resolution (3.0 Å) has been reported earlier for wild-type *Ho*BGLA_*PET*_ (Protein Data Bank, PDB, code 3TA9; Kori et al. 2011). *Ho*BGLA was concentrated to 50 mg/mL in a solution containing 20 mM HEPES pH 7.0 and 150 mM NaCl. Crystallization screening was performed using the sitting-drop vapor diffusion method in 96-well screening plates (Corning 3550 96-well sitting drop plate) and dispensed by a mosquito®Crystal robotics (TTP Labtech) with drop size of 300 nL and protein-to-reservoir ratios of 1:1, 1:2, and 2:1. Solutions were from the commercial screens PACT Suite (Qiagen), JCSG Suite (Qiagen), and Crystal Screen HT (Hampton Research). Initial screen hits were optimized using 24-well plates (INTELLI-PLATE™24, Art Robbins Instrument). Well-diffracting crystals were obtained in 0.1 M sodium cacodylate in the pH range 5.5–6.5, polyethylene glycol (PEG) 3350 in the concentration range 25–30 % (*w/v*), and in the presence of either MgCl_2_, CsCl_2_ or sodium acetate. *Ho*BGLA structures were determined in complex with the ligands thiocellobiose (TCB), (thiocellobiose, 2-deoxy-2-fluoro-d-glucose (*2F*Glc; Sigma-Aldrich Co. LLC., USA; Cat. N^o^. F5006-25 mg), and cellobionolactam. The ligand complexes of *Ho*BGLA were produced by immersing the crystals briefly in reservoir solution containing the ligand at saturating concentration, followed by vitrification in liquid nitrogen.

Crystals of *Ho*BGLA were soaked in the presence of TCB, *2F*Glc, or cellobionolactam, an inhibitor of some glycoside hydrolases. In the case of the cellobionolactam-soaked crystal, the ligand was cleaved leaving only glucose (Glc) bound in the active site. We will hereafter refer to this complex as glucose rather than a cellobionolactam complex. The optimized crystallization conditions for the *Ho*BGLA-ligand complexes were wild-type *Ho*BGLA–thiocellobiose (TCB), 0.1 M sodium cacodylate pH 6.5, 0.1 M CsCl_2_, and 26 % PEG 3350; *Ho*BGLA–*2F*Glc, 0.1 M sodium cacodylate pH 6.5, 0.17 M sodium acetate, and 30 % (*w/v*) PEG 3350; and *Ho*BGLA–Glc, 0.1 M sodium cacodylate pH 5.5, 0.3 M MgCl_2_, and 25 % (*w/v*) PEG 3350. All crystals were grown at room temperature.

X-ray intensity data were collected at 100 K using synchrotron radiation at the following ESRF (Grenoble, France) beamlines: *Ho*BGLA–TBC, ID23-2; *Ho*BGLA–*2F*Glc and *Ho*BGLA–Glc at ID14-4. All data processing and scaling were performed using the XDS package (Kabsch 1993). Structure determination was performed by molecular replacement with PHASER implemented in the PHENIX suite (Adams et al. 2010) using the previously deposited 3.0-Å model of wild-type *Ho*BGLA_*PET*_ (PDB code 3TA9; Kori et al. 2011) as search model. Model building was performed using COOT (Emsley and Cowtan 2004) and O (Jones et al. 1991), and refinement using the PHENIX software package (Adams et al. 2010). Figures showing structural information were prepared with PyMOL (DeLano Scientific LLC, Palo Alto, CA, USA). Coordinates and structure factors are available in the Protein Data Bank database (http://www.rcsb.org) with the following PDB accession numbers: recombinant wild-type *Ho*BGLA–TCB, 4PTV; *Ho*BGLA–*2F*Glc, 4PTW; and *Ho*BGLA–Glc, 4PTX.

## Results

### Biochemical characterization of wild-type and mutant HoBGLA

*Ho*BGLA was typically expressed in yields of 1.5 mg/L culture and purified to high homogeneity and monodispersity. The substrate specificity of purified *Ho*BGLA was determined towards various aryl glycosides (*o*NPGal, *p*NPGal, *o*NPGlc, and *p*NPGlc) and disaccharides (cellobiose and lactose), and kinetic constants were determined for these substrates (Table 1). The apparent turnover values (*k*_cat,app_) were calculated using the experimentally determined *v*_max_ values and a molecular mass of 53 kDa for the enzyme. The apparent catalytic efficiencies (*k*_cat,app_/*K*_m_) indicate that the glucopyranosides are better substrates than their galactopyranoside counterparts. This is mainly based on the lower Michaelis constant *K*_m_ for the glucose-containing substrates (*o*NPGlc, *p*NPGlc, and cellobiose) as compared to the corresponding galactose-containing molecules (*o*NPGal, *p*NPGal, and lactose).

**Table 1:** Kinetic parameters for *β*-glucosidase and *β*-galactosidase activities of wild type and mutant *Ho*BGLA

*β*-glucosidase activity^1^									
	d-cellobiose			*o*NPGlc			*p*NPGlc		
	*k*_cat,app_ (s^−1^)	*K*_m_ (mM)	*k*_cat,app_/*K*_m_ (mM^−1^ s^−1^)	*k*_cat,app_ (s^−1^)	*K*_m_ (mM)	*k*_cat,app_/*K*_m_ (mM^−1^ s^−1^)	*k*_cat,app_ (s^−1^)	*K*_m_ (mM)	*k*_cat,app_/*K*_m_ (mM^−1^ s^−1^)
Wild-type	366	25.4 ± 2.8	14.4	94.6	0.386 ± 0.002	245	55.2	0.296 ± 0.001	187
E354Q	n.d.	n.d.	n.d.	–	–	–	–	–	–
E166Q	6.8	27.7 ± 6.4	0.25	–	–	–	–	–	–
E408Q	24	19.4 ± 2.3	1.24	–	–	–	–	–	–
*β*-galactosidase activity^2^									
	d-lactose			*o*NPGal			*p*NPGal		
	*k*_cat,app_ (s^−1^)	*K*_m_ (mM)	*k*_cat,app_/*K*_m_ (mM^−1^ s^−1^)	*k*_cat,app_ (s^−1^)	*K*_m_ (mM)	*k*_cat,app_/*K*_m_ (mM^−1^ s^−1^)	*k*_cat,app_ (s^−1^)	*K*_m_ (mM)	*k*_cat,app_/*K*_m_ (mM^−1^ s^−1^)
Wild-type	231	154 ± 24	1.50	288	6.35 ± 0.8	45.3	156	8.34 ± 0.61	18.7
E354Q	n.d.	n.d.	n.d.	–	–	–	–	–	–
E166Q	0.47	36.7 ± 3.8	0.013	–	–	–	–	–	–
E408Q	76	111 ± 7.3	0.68	–	–	–	–	–	–

^1^The *β*-glucosidase activity was measured using the natural substrate d-cellobiose, as well as the chromophoric substrates *o*NPGlc and *p*NPGlc. For the chromophoric substrates, kinetic parameters were measured only for the wild-type enzyme

^2^The *β*-galactosidase activity was measured using d-lactose, and the chromophoric substrates *o*NPGal and *p*NPGal. For the chromophoric substrates, kinetic parameters were measured only for the wild-type enzyme

*n.d*. no activity could be determined under standard assay conditions

**Fig. 1:**
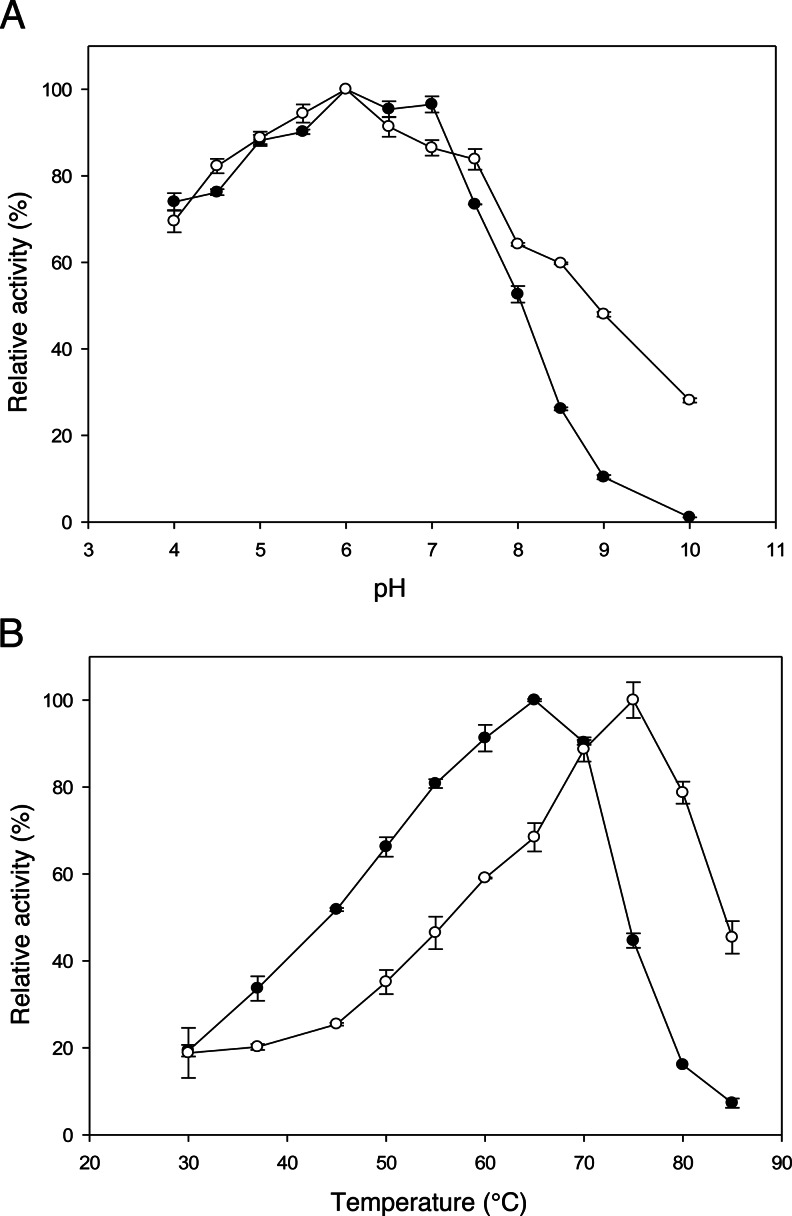
Effect of pH and temperature on the β-galactosidase activity of *Ho*BGLA. The dependency of *Ho*BGLA β-galactosidase activity on pH (**a**) and temperature (**b**) optimum using *o*NPGal and d-lactose as substrates. Symbols: (*black circle*) *o*NPGal and (*white circle*) d-lactose. The standard β-galactosidase assay was used, and the activity analyzed using *o*NPGal in the pH range 4–10 using Briton–Robinson buffer (see Materials and methods for details). The temperature optima for activity with lactose and *o*NPGal as substrates were determined in the temperature range 30–85 °C, in 20 mM HEPES and 150 mM NaCl (pH 7.0)

Aiming at a dairy application for this enzyme, *o*NPGal and lactose were used as substrates to determine the optimal temperature and pH of *Ho*BGLA activity. The pH optimum for the *β*-galactosidase activity was studied over a pH range of 4.0 to 10.0 and at 30 °C. Maximal *β*-galactosidase activity was obtained at pH 6.0 for both substrates. When *o*NPGal was used as a substrate, *Ho*BGLA showed more than 80 % of its maximum activity in the pH range of pH 5.0–7.0 (Fig. 1a), while when using lactose as a substrate, the enzyme showed more than 80 % of its maximum activity in the pH range of pH 4.5–7.5. In the pH range tested (pH 4–10), the enzyme was inactivated at pH values above 8.0 (data not shown).

The temperature optimum for the *β*-galactosidase activity of *H. orenii Ho*BGLA was determined over the temperature range 30–85 °C. Maximal *β*-galactosidase activity was obtained at 65 and 75 °C for *o*NPGal and lactose, with specific activity values of 304 U_oNPGal_/mg and 262 U_Lac_/mg, respectively (Fig. 1b). The relative activity of *Ho*BGLA was higher for lactose than for *o*NPGal at temperatures above 75 °C. Furthermore, the enzyme retained 90 % of its activity after 3 h of incubation at 65 °C (Fig. S1). The enzyme showed half-life times of activity (τ_1/2_) of 18 and 6 h at 65 and 70 °C, respectively. We also investigated the effect of various metal ions on thermal stability of *Ho*BGLA *β*-galactosidase activity, however, only Mg^2+^ produced a positive effect. When 1 mM Mg^2+^ was added, thermal stability of the enzyme as expressed by τ_1/2_ of *Ho*BGLA activity slightly increased to 24 h at 65 °C and 9 h at 75 °C (Fig. S1). The stabilizing effect of Mg^2+^ on the *β*-galactosidase activity of this *β*-glucosidase is in agreement with what we have observed previously for true *β*-galactosidases (Nguyen et al. 2006; Iqbal et al. 2011).

The kinetic constants for cellobiose and lactose as substrates are summarized in Table 1 for wild-type and mutant *Ho*BGLA. As expected, the replacement of the catalytic nucleophile by a glutamine side chain (E354Q) results in a catalytically incompetent variant without detectable activity on either cellobiose or lactose. With cellobiose as substrate, replacing the acid/base catalyst by a glutamine (E166Q) does not alter the *K*_m_ value, but reduces the *k*_cat,app_ value 54-fold, giving a specificity constant, *k*_cat,app_/*K*_m_, that is 59-fold lower than for the wild type. With lactose as the substrate, the *K*_m_ value for E166Q improves slightly relative to the wild type (fourfold reduction), but at the expense of a 500-fold reduction in the *k*_cat,app_ value, resulting in more than 100-fold lower specificity constant value. Glu408 is proposed to participate in substrate binding, and replacement by a glutamine side chain has a little effect on *K*_m_ (1.3-fold reduction for both cellobiose and lactose), whereas *k*_cat,app_ is reduced 15-fold for cellobiose and 3-fold for lactose.

### Galacto-oligosaccharide synthesis

**Fig. 2:**
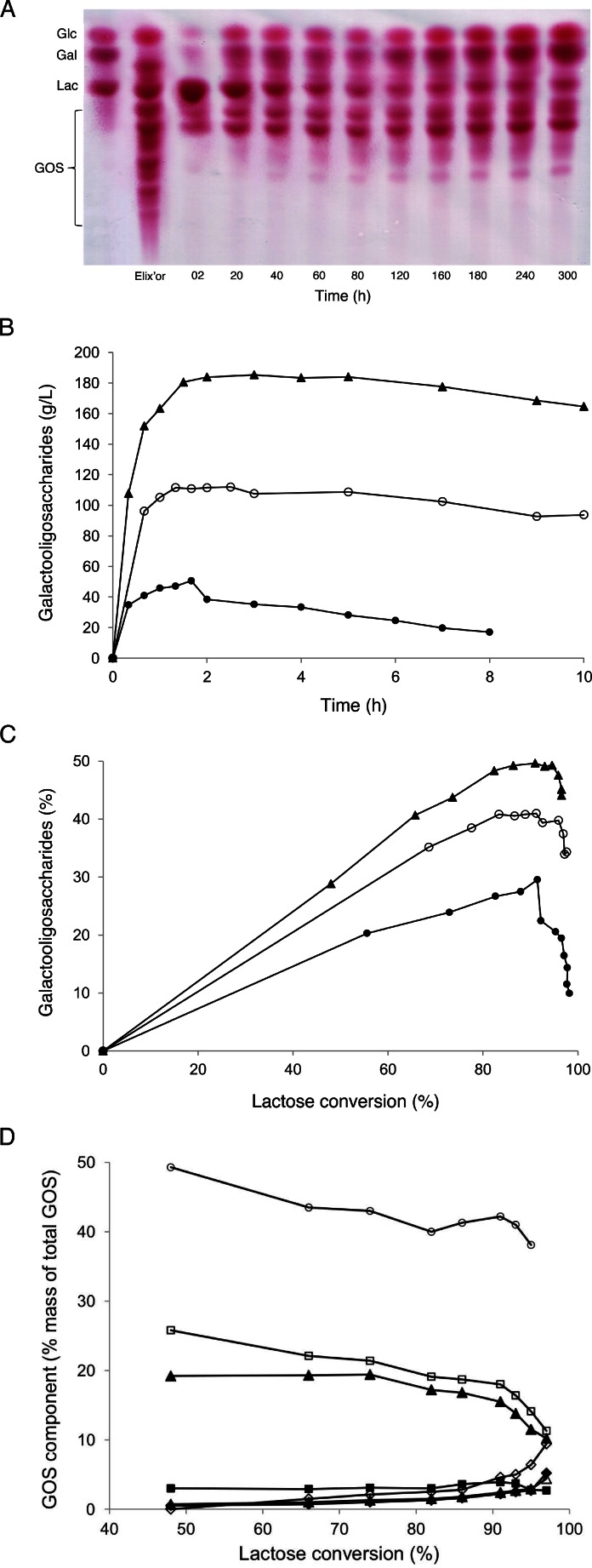
Transglycosylation products from lactose hydrolysis. **a** Hydrolysis of lactose catalyzed by *Ho*BGLA as analyzed by TLC on preactivated silica plates (eluent: *n*-butanol–*n*-propanol–ethanol–water = 2:3:3:2). The reaction was carried out at 70 °C with an initial lactose concentration of 205 g/L in 20 mM HEPES and 150 mM NaCl (pH 7.0), containing 1 mM MgCl_2_ and 12.0 U_*o*NP_/mL of *Ho*BGLA. Samples were withdrawn at regular time intervals during the reaction. A commercially available GOS preparation, Elix’or (Friesland Foods Domo), was used for comparison. **b** Time-course of total GOS production catalyzed by wild-type *Ho*BGLA. The reaction was performed at 70 °C, 300 rpm at various initial lactose concentrations (200, 300, and 400 g/L) in HEPES and 150 mM NaCl (pH 7.0), containing 1 mM MgCl_2_ using 12.0 U_*o*NP_/mL_._ Symbols: (*black circle*) 200 g/L initial lactose concentration; (*white circle*) 300 g/L initial lactose concentration; and (*black triangle*) 400 g/L initial lactose concentration. **c** GOS yield (% of total mass) at different lactose conversion catalyzed by wild-type *Ho*BGLA. The reaction was performed at 70 °C, 300 rpm at various initial lactose concentrations (200, 300, and 400 g/L) in HEPES and 150 mM NaCl (pH 7.0), containing 1 mM MgCl_2_ using 12.0 U_*o*NP_/mL. Symbols: (*black circle*) 200 g/L initial lactose concentration; (*white circle*) 300 g/L initial lactose concentration; and (*black triangle*) 400 g/L initial lactose concentration. **d** Individual GOS components produced by the transgalactosylation reaction of wild-type *Ho*BGLA using lactose as substrate. Symbols: (*white diamond*) d-Gal*p*-(1→6)-d-Glc; (*black diamond*) d-Gal*p*-(1→6)-d-G*al*; (*delta*) d-Gal*p*-(1→*3*)-d-G*al*; (*black triangle*) d-Gal*p*-(1→*3*)-d-G*lc*; (*white square*) d-Gal*p*-(1→*3*)-d-*Lac*; (*black square*) d-Gal*p*-(1→*4*)-d-*Lac*; (*white circle*) d-Gal*p*-(1→*6*)-d-*Lac*

A spectrum of different galacto-oligosaccharides (GOS) was produced during conversion of lactose at 70 °C with an initial lactose concentration of 205 g/L catalyzed by *Ho*BGLA as analyzed by thin layer chromatography (TLC) (Fig. 2a). It was shown that lactose was cleaved and GOS was formed soon after the reaction was started. Subsequently, the influence of the initial lactose concentration on GOS production using *Ho*BGLA was investigated. For initial lactose concentrations of 200, 300, and 400 g/L, the maximum GOS yields were 51, 112, and 185 g/L (Fig. 2b), or approximately 30, 41, and 50 % (Fig. 2c). These amounts were obtained within 2 to 3 h of reaction at 91 % lactose conversion.

Individual GOS can be separated effectively using a Carbopac PA1 column for HPAEC-PAD (Fig. S2). It was possible to identify the main products of the transgalactosylation reaction of *Ho*BGLA when lactose was the substrate (Fig. 2d). The predominant oligosaccharide product was identified as *β*-d-Gal*p*-(1→6)-d-Lac, accounting for approximately 42 % of the total GOS formed at maximum GOS yield. *β*-d-Gal*p*-(1→3)-d-Lac was identified as the second predominant transferase product at the maximum total GOS yield point, contributing approximately 18 % of the total GOS formed. Other identified products that are formed to lesser amounts include *β*-d-Gal*p*-(1→3)-Glc, *β*-d-Gal*p*-(1→3)-Gal, *β*-d-Gal*p*-(1→6)-Gal, *β*-d-Gal*p*-(1→6)-Glc, and *β*-d-Gal*p*-(1→4)-Lac.

### Overall structure of HoBGLA

Data collection and refinement statistics for the three wild-type *Ho*BGLA models are presented in Table S1. As reported previously (Kori et al. 2011), the overall structure of the 451-residue large *Ho*BGLA displays the typical (β/α)_8_ TIM-barrel fold adopted by retaining GH1 enzymes. For all three models, the residues Met1-Ala2-Lys3 and the three C-terminal residues E449-Ala450-Asn451 are missing due to local disorder and lack of interpretable electron density at the N- and C-terminus, respectively. Here, we focus the structural description on the details of ligand binding in the active site.

### Structure of the HoBGLA-thiocellobiose complex

**Fig. 3:**
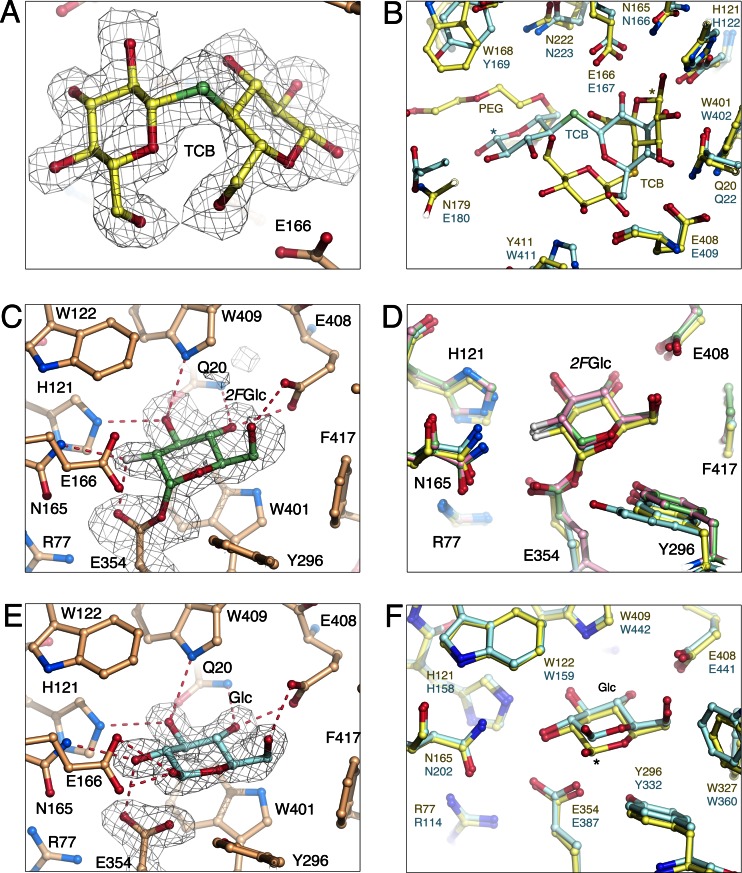
Structural details of ligand binding. **a** Binding of thiocellobiose to *Ho*BGLA with superimposed 2*F*_o_–*F*_c_ electron density. **b** Overlay of the *Ho*BGLA-TCB complex (*yellow*) with *P. polymyxa* β-glucosidase B (PDB code 2O9R; Isorna et al. 2007) in blue color. The *asterisk* denotes the C1 position of the reducing end glucosyl unit. **c** Binding of 2-deoxy-2-fluoro-d-glucose to *Ho*BGLA with superimposed positive difference omit Fourier map contoured at 2.5σ. **d** Overlay of the *Ho*BGLA-*2F*Glc complex (*yellow*) with those of rice Os4BGlu12 (*blue*; PDB code 3PTM; Sansenya et al. 2011), wheat β-glucosidase (*green*; PDB code 3AIR; Sue et al. 2011), and rye β-glucosidase (*pink*; PDB code 3AIW; Sue et al. 2011). **e** Binding of β-d-glucose to *Ho*BGLA with superimposed positive difference omit Fourier map contoured at 5σ. **f** Overlay of the *Ho*BGLA-Glc complex (*yellow*) with a β-glucosidase B from an uncultured bacterium (PDB code 4HZ8; Nam et al. 2010) in *blue* color. The *asterisk* denotes the C1 position in the glucosyl unit. The pictures were made using the program PyMOL (De Lano 2002)

The crystal structure of *Ho*BGLA in complex with thiocellobiose, *Ho*BGLA–TCB, was determined at 1.85 Å resolution, and shows well-defined electron density for the TCB ligand (Fig. 3a). To date, only one TCB complex of a GH1 glycoside hydrolase is reported, namely that of β-glucosidase B from *Paenibacillus polymyxa* (*Pp*BGLB; PDB code 2O9R; Isorna et al. 2007). Considering the overall similarity of the active site in *Ho*BGLA and *Pp*BGLB, the striking difference in TCB binding was unexpected (Fig. 3b). In *Pp*BGLB, TCB binds with the nonreducing-end glucosyl unit in subsite −1 and the reducing-end glucosyl in +1, similar to what is expected based on BGL complexes of longer cellooligomers, e.g., rice *β*-glucosidase BGlu1 (PDB code 3F5K; Chuenchor et al. 2011). In *Ho*BGLA, however, the TCB molecules folds into an unusual conformation to place the reducing end O1 atom within short hydrogen-bonding distance of the catalytic nucleophile Glu354 Oε2 atom (Table S2). The reason is most likely a bound PEG molecule occupying part of the +1 subsite, +2, +3, and part of +4 (number of subsites inferred from PDB code 3F5K), which competes with TCB for the +1 site and effectively forces it to adopt a different conformation. The binding of the PEG molecule traces out the better part of the substrate-binding cleft showing that *Ho*BGLA can indeed accommodate extended molecules, which is relevant for synthesis of longer GOS products.

In rice BGlu1, an extended loop comprising residues 322–335 delineates the far “plus end” of the binding cleft and its tip folds to form one side of the substrate-binding cleft. The corresponding loop in *Ho*BGLA is considerably shorter (residues 303–308), which provides more space in this region of the cleft. Consequently, the PEG molecule is allowed to bind differently in +4 than the corresponding glucosyl unit in cellopentaose bound to BGlu1 (Fig. S3). We predict that an oligosaccharide longer than four sugar units, i.e., binding beyond subsite +3, could be bound to *Ho*BGLA either as observed for the cellopentaose in BGlu1, or possibly, as observed for PEG in the TCB complex. Based on the binding of PEG to *Ho*BGLA, and the 3F5K structure, the following residues may be part of the putative binding sites +3 and +4: Val314, Leu242, Tyr245, Phe177, Asn308, Asp307, and Glu313.

Thus, despite this TCB binding mode being an obvious artifact, the importance of the complex is twofold: firstly, it allows us to verify an extended substrate-binding region where longer transglycosylation products may bind; and secondly, it provides additional information regarding possible conformers for TCB, which is valuable considering that only four TCB-bound structures exist in the Protein Data Bank, neither of which displays this conformation.

### Structure of the covalent HoBGLA-2FGlc complex

The attachment of an electronegative fluorine atom close to the reacting carbon atom serves to destabilize the oxocarbenium ion-like transition state and reduces the reaction rates in both the glycosylation and deglycosylation steps of the retaining reaction. To allow accumulation and trapping of the fluoroglucopyranosyl-nucleophile intermediate by making the deglycosylation step rate-limiting, a good leaving group (a highly reactive aglycon) is attached to the substrate to selectively slow down the breakdown of the intermediate relative to the rate of formation (Withers et al. 1987, 1988).

The 2.0 Å structure of the 2-deoxy-2-fluoroglucopyranosyl-*Ho*BGLA intermediate was obtained by soaking crystals with only *2F*Glc and not with an activated compound such as 2,4-dinitrophenyl 2-deoxy-2-fluoro-*β*-d-glucoside (DNP-*2F*Glc) or 2-deoxy-2-fluoro-*β*-d-glucosyl fluoride. Nonetheless, the catalytic nucleophile appears to be labeled by *2F*Glc (Fig. 3c; Table S2) in both molecules of the asymmetric unit with covalent-bond (Glu354 Oε1–*2F*Glc C1) distances of 1.60 and 1.58 Å, respectively. It is likely that the covalent complex is a result of reverse hydrolysis. Based on the elongated bond distance compared with that expected for a stable covalent intermediate (about 1.35 to 1.45 Å), the structure may reflect a somewhat destabilized covalent complex. However, there are no obvious alternative orientations of either the *2F*Glc molecule or the Glu354 side chain. It is also clear from the electron density that the equatorial O1 hydroxyl group for *2F*Glc has been removed. The *2F*Glc-labeled nucleophile complex of *Ho*BGLA is very similar to that of other *2F*Glc complexes of wild-type GH1 BGLs (PDB codes: 3PTM, 3AIR, 3AIW, 3GNR, 2RGM, 2JIE, 1UWS, 1OIN, and 1W4I), of which the *β*-glucosidase complexes from rice (PDB code 3PTM), wheat (PDB code 3AIR), and rye (PDB code 3AIW) are nearly identical (Fig. 3d).

In subsite −1 of the active site, the catalytic residues Glu354 (nucleophile) and Glu166 (acid/base catalyst) are situated near Glu408. The Glu408 Oε1 and Oε2 oxygen atoms participate in substrate binding by offering two hydrogen bonds to O4 in *2F*Glc. Considering that a glutamine can provide the same hydrogen bonds, the 15-fold decrease in *k*_cat,app_ for the E408Q mutant is probably due to perturbation of the electrostatic environment of the active site. Trp401 provide hydrophobic stacking with the glucosyl residue in this subsite and additional protein-sugar hydrogen bonds are formed by Gln20, Trp409, His121, Asn165, and Glu354 (Fig. 3c; Table S2).

The Cremer-Pople puckering parameters for *2F*Glc were analyzed using the Cremer-Pople parameter calculator (http://www.ric.hi-ho.ne.jp/asfushi/). The puckering parameters [φ = 103.4°, θ = 7.0°, Q = 0.64] are consistent with a ^4^ *C*_1_ chair with a slight distortion in the direction of a ^2,5^ *B* boat/^5^ *S*_1_ skew boat. For comparison, puckering parameters for the three structurally most similar covalent *2F*Glc complexes are: 3PTM (φ = 195.0°, θ = 3.7°, Q = 0.60); 3AIR (φ = 255.1°, θ = 13.2°, Q = 0.56); and 3AIW (φ = 36.2°, θ = 4.4°, Q = 0.65).

### Structure of the HoBGLA-glucose complex

The crystal structure of *Ho*BGLA in complex with glucose, representing the post-hydrolysis state, was determined and refined at 1.80 Å resolution (Fig. 3e; Table S2). Of the many previously reported crystal structures of GH1 BGLs in complex with glucose, that of a BGL from an uncultured bacterium (PDB codes 4HZ7 and 4HZ8; Nam et al. 2010) is the most similar with respect to structural details of glucose binding (Fig. 3f). The glucose product interacts intimately with protein to allow all its exocyclic hydroxyl groups to be positioned within hydrogen-bonding distance to the primary shell of protein side chains in subsite −1. Despite being non-covalently bound, the protein interactions made by the glucose molecule are identical to those observed for the covalently linked *2F*Glc, the exception being that the acid/base catalyst Glu166 forms two hydrogen bonds to the Glc O1 hydroxyl group (Fig. 3e; Table S2).

The Cremer-Pople puckering parameters for glucose bound to *Ho*BGLA are (φ = 237.9°, θ = 15.7°, Q = 0.57), consistent with a ^4^ *C*_1_ chair somewhat distorted toward a ^1,4^ *B* boat. The puckering parameters for the structurally most similar glucose complex are 4HZ8 (φ = 270.8°, θ = 18.4°, Q = 0.60) where the glucose molecule is distorted from the relaxed ^4^ *C*_1_ chair in the direction of a ^1^ *S*_5_ skew boat.

## Discussion

A number of retaining GH1 BGLs are capable of catalyzing both hydrolysis and transglycosylation reactions; however, a little is known about the factors that determine the balance between the two activities (Teze et al. 2014). Owing to the practical applications of GOS production from hydrolysis of milk lactose, several attempts have been made to engineer BGLs towards *sufficient* transglycosylation activity while keeping the hydrolysis activity at a minimum, or towards a *higher* transglycosylation-to-hydrolysis ratio compared with the wild type through mutations targeting either the aglycon or glycon binding site of the enzyme (Hansson et al. 2001; Feng et al. 2005).

The GH1 *β*-glucosidase *Ho*BGLA is produced by the thermophilic bacterium *Halothermothrix orenii*, a bacterium that grows optimally at 60 °C with NaCl concentrations ranging between 5 and 10 % (Cayol et al. 1994). In this study, we show that *Ho*BGLA displays promising characteristics for GOS production compared with GOS-producing BGLs reported to date. *Ho*BGLA hydrolyzes both β-glucosides such as cellobiose, and β-galactosidases such as lactose. Based on our kinetic analysis, β-glucosides are the preferred substrates, and hence, the name *β*-glucosidase can be used for the enzyme. *Ho*BGLA is a nonspecific BGL with mixed activities for different substrates, and shows prominent activity with various galactosides. The apparent Michaelis constant of *Ho*BGLA for lactose is relatively high compared to the values reported for some commonly used commercial enzymes (*A. oryzae*, 36–180 mM; *A. niger*, 54–99 mM; *K. fragilis*, 15–52 mM; and *K. lactis*, 35 mM; Jurado et al. 2002; de Roos 2004). Most of these commercial enzymes are from mesophilic sources, whereas *Ho*BGLA is thermophilic and the kinetic constants were measured at 65 °C. It is known that the apparent strength of substrate binding decreases with increasing temperature. A clear disadvantage of high *K*_m,Lac_ values is that complete substrate conversion in a single-stage continuous tank reactors is difficult to achieve. Nonetheless, a number of favorable characteristics make *Ho*BGLA an attractive biocatalyst for lactose conversion: (i) pH optimum of about 6 for lactose hydrolysis; (ii) the broad optimal stability over the pH range of 4.5 to 7.5; (iii) a temperature optimum in the range 65–70 °C; and (iv) its thermostability within the aforementioned temperature range.

A major drawback of using mesophilic biocatalysts in industrial processes is the risk of microbial contamination. Working under sterile conditions requires special equipment and extra process steps leading to additional costs. Apart from the microbial quality of the raw materials, reaction temperature and conversion rate are important parameters to overcome these problems. *Ho*BGLA is thus a promising candidate for lactose hydrolysis and GOS formation at 65–70 °C. The highest total GOS yield of approximately 50 % was obtained in discontinuous conversion reactions with an initial lactose concentration of 400 g/L. This value lays in the upper range of enzymatic GOS productivity reported so far (Park and Oh 2010). The major products are β-d-Gal*p*-(1→6)-d-Lac and β-d-Gal*p*-(1→3)-d-Lac, indicating that lactose is a far better galactosyl acceptor than glucose and galactose, and that *Ho*BGLA has a high specificity for forming β-1→6 and β-1→3 linkages.

Although the glucose inhibition profile of *Ho*BGLA has not been investigated specifically, the achievement of >97 % lactose conversion within a short period of time indicates high tolerance to product inhibition. Based on the comparably good thermal stability and the high transgalactosylation activity, this enzyme should be useful for the efficient hydrolysis of lactose in milk and whey, as well as for the production of lactose-derived oligosaccharides. In addition, this non-specific BGL can be used in enzyme systems for degradation of cellulose or cellodextrins during growth on lignocellulose.

**Fig. 4:**
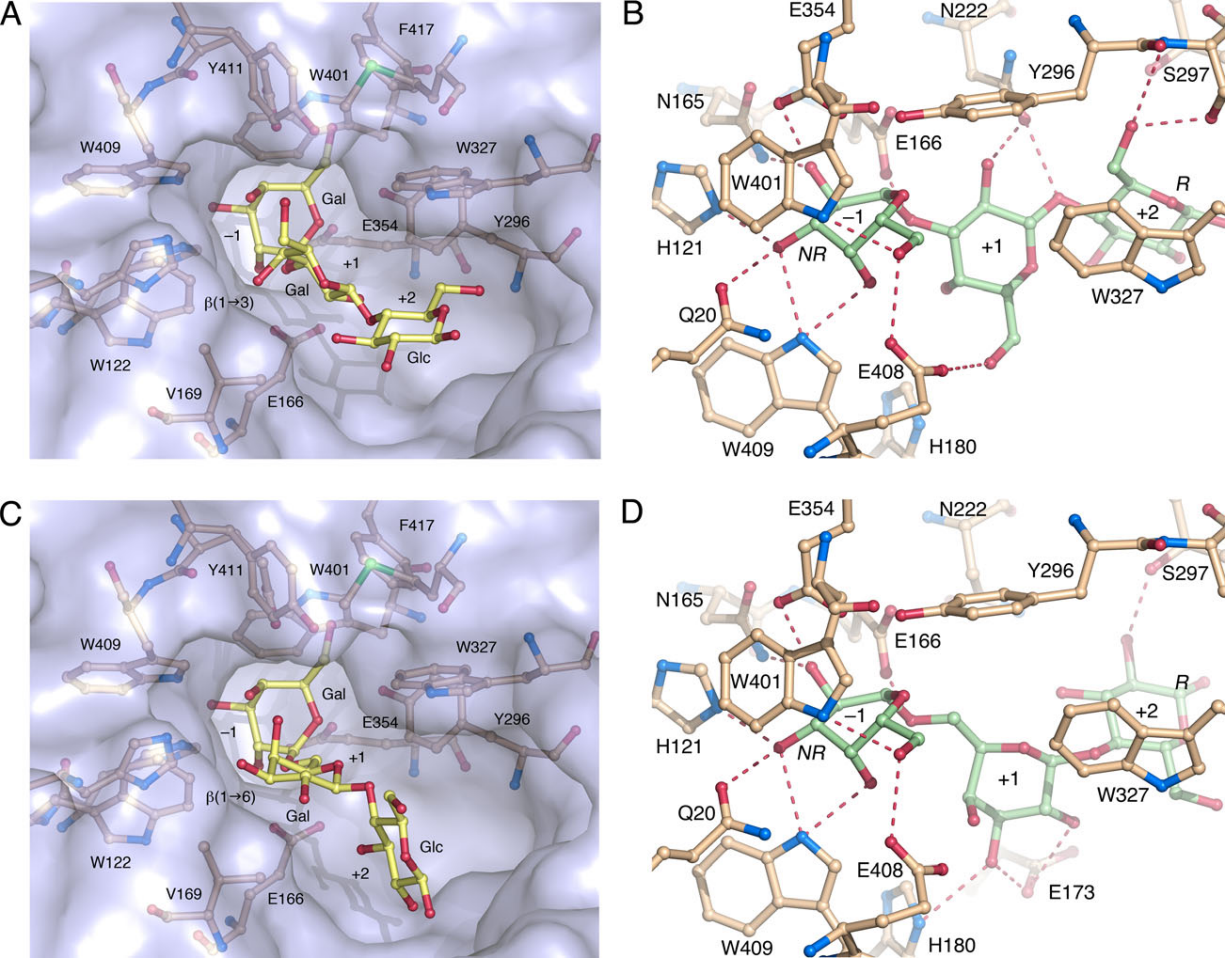
Theoretical modeling of the major trisaccharide GOS products 3GALA and 6GALA. **a** Overview of modeled 3GALA in *Ho*BGLA in the substrate-binding cleft of *Ho*BGLA, and **b** details of predicted protein-sugar interactions in subsites −1, +1, and +2. **c** Overview of 6GALA binding and (**cus**) details of predicted protein-sugar interactions in subsites −1, +1, and +2. The ^1,4^ *B* boat conformation of the galactosyl unit in subsite −1 for 3GALA and 6GALA was based on the glucose conformer observed in the cellopentaose complex of rice BGlu1 (PDB code 3F5K; Chuenchor et al. 2011). The subsites are denoted −1, +1, and +2, and the reducing and non-reducing end sugar units are marked by R and NR, respectively. Hydrogen bonds are shown as *dashed red lines*. The protein–sugar interactions are also listed in Table S3

In order to investigate how well the major GOS products, β-d-Gal*p*-(1→3)-d-Lac and β-d-Gal*p*-(1→6)-d-Lac, can be accommodated in the *Ho*BGLA substrate-binding cleft, we modeled the two trisaccharides 3GALA and 6GALA manually. To guide modeling, the *Ho*BGLA complexes and the cellopentaose (G5) complex of rice BGlu1 was analyzed in detail (PDB code 3F5K; Chuenchor et al. 2011). The BGlu1-G5 complex shows the non-reducing end glucosyl unit in subsite −1 adopting a boat whereas the glucosyl units in subsites +1 to +4 adopt the relaxed ^4^ *C*_1_ chair conformation. The two GOS trisaccharides were modeled to mimic closely the glucosyl-binding modes in subsites −1 and +1 of BGlu1, but with a non-reducing end galactose unit of 3GALA and 6GALA in subsite −1. In 3GALA, the skew-boat galactose residue in −1 is linked at C1 to C3 of the galactosyl unit (in subsite +1) of lactose by a β-1,3-glycosidic bond (Fig. 4a, b). In 6GALA, the linkage between galactose and lactose is instead a β-1,6-glycosidic bond between C1 of the skew-boat galactose (subsite −1) and the exocyclic C6 carbon of the +1 subsite galactosyl unit in lactose (Fig. 4c, d). The modeling suggests that both 3GALA and 6GALA are well accommodated in the binding cleft of *Ho*BGLA such that the non-reducing end galactosyl unit is bound in subsite −1, the second galactosyl unit in subsite +1 and the reducing-end glucosyl unit in subsite +2. These models provide an initial structural framework for further design of mutants with improved transgalactosylation and GOS production; however, the precise structural determinants will depend on which GOS is to be synthesized. There are several examples of engineering of BGLs to achieve higher transglycosylation efficiency for certain products. In the case of *Ho*BGLA, the inherently high stability and transgalactosylation activity provide an ideal starting point for enzyme engineering towards improved 3GALA/6GALA yields or other GOSs.

Examples of BGLs that have been engineered towards improved GOS yield are from the hyperthermophilic archaea *Pyrococcus furiosus* (CelB) and *Sulfolobus solfataricus* P2 (LacS). For CelB, the mutant F426Y showed an oligosaccharide yield of 45 % (*w/w*) compared to 40 % for the wild type (Hansson et al. 2001). This mutant had improved affinity for galactosidases as judged by a decrease in *K*_m_ from 2.3 to 0.9 mM (Kaper et al. 2000). In the case of LacS, two single amino-acid replacements F359Q and F441Y (F426Y in CelB) resulted in an increase in GOS yield from 51 % for the wild type to 58 and 62 %, respectively (Wu et al. 2013). Unfortunately, no data were reported for the double mutant. Although the precise mutations may not be useful for improving the GOS yield by *Ho*BGLA, they can provide guidance on suitable future engineering strategies for improved GOS yields from *Ho*BGLA transgalactosylation.

## Electronic supplementary material

Below is the link to the electronic supplementary material.

ESM 1 (PDF 1360 kb)
