# Supplementary material for: Biochemical and structural characterization of a thermostable β-glucosidase from Halothermothrix orenii for galacto-oligosaccharide synthesis
Source: Appl Microbiol Biotechnol. 2014 Aug 31;99(4):1731–44. doi: 10.1007/s00253-014-6015-x (PMC4322223; doi:10.1007/s00253-014-6015-x)
Supplement: Supplementary file 1 — (PDF 1360 kb) [file 253_2014_6015_MOESM1_ESM.pdf]

Applied Microbiology and Biotechnology

Supplementary material

Biochemical and structural characterization of a thermostable *β*-glucosidase from *Halothermothrix orenii* for galacto-oligosaccharide synthesis

Noor Hassan[1†], Thu-Ha Nguyen[2†], Montira Intanon[2], Lokesh D. Kori[3,4], Bharat K. C. Patel[3], Dietmar Haltrich[2], Christina Divne[1,5], Tien Chye Tan[1,5*]

[1] KTH Royal Institute of Technology, School of Biotechnology, Albanova University Center, Roslagstullsbacken 21, S-10691 Stockholm, Sweden

[2] BOKU University of Natural Resources and Life Sciences, Food Biotechnology Laboratory, A-1190 Vienna, Austria

[3] Griffith University, School of Biomolecular and Physical Sciences, Microbial Gene Research and Resources Facility, Brisbane, QLD 4111, Australia

[4] Baylor College of Medicine, Biochemistry and Molecular Biology, Houston, TX, USA 77030

[5] Karolinska Institute, Department of Medical Biochemistry and Biophysics, Scheelelaboratoriet, Scheeles väg 2, S-17177 Stockholm, Sweden

† Joint first authorship

* Corresponding author; *E-mail address:* tantc@kth.se (TC Tan); *Address:* KTH Royal Institute of Technology, School of Biotechnology, Albanova University Center, Roslagstullsbacken 21, S-10691 Stockholm, Sweden; Phone: +46-73-8425695.

Table S1. Data collection and crystallographic refinement statistics for wild-type *Ho*BGLA ligand complexes

Data collection[1]			
Protein variant	*Ho*BGLA–TCB	*Ho*BGLA–*2F*Glc	*Ho*BGLA–Glc
Space group / mol. per a.s.u.	$P2_12_12_1$ / 2	$P2_12_12_1$ / 2	$P2_12_12_1$ / 2
Cell constants *a*, *b*, *c* (Å)	87.75, 99.07, 108.09	88.98, 97.54, 109.62	89.45, 97.88, 109.88
Beamline, λ (Å)	ESRF ID23-2, 0.87260	ESRF ID14-4, 0.93935	ESRF ID14-4, 0.93935
Resolution range (Å)	49.53–1.85 (1.90–1.85)	47.78 –2.00 (2.10–2.00)	46.81 –1.80 (1.90–1.80)
Unique reflections	80,889 (6,154)	64,629 (8,644)	88,436 (12,942)
Multiplicity	8.2 (8.3)	7.3 (7.5)	7.4 (7.6)
Completeness (%)	99.9 (99.8)	99.3 (98.8)	98.4 (97.2)
$<I / \sigma I>$	12.4 (1.2)	12.4 (1.9)	14.0 (1.4)
R_{sym}[2]	0.253 (1.866)	0.157 (1.310)	0.181 (2.228)
R_{meas}	0.270 (1.991)	0.169 (1.407)	0.194 (2.391)
CC(1/2)[3]	99.3 (50.6)	99.6 (63.4)	99.8 (53.0)
Crystallographic refinement			
Resolution range (Å)	49.53–1.85 (1.90 –1.85)	47.78–2.00 (2.05 – 2.00)	46.81–1.80 (1.84 – 1.80)
Completeness, all % (outer bin)	99.5 (100)	99.3 (99.0)	98.4 (97.0)
R_{factor}[4]/work reflections, all	0.196 / 80,626	0.177 / 64,627	0.183 / 88,435
R_{free}/free reflections, all	0.227 / 2,006	0.235 / 2,000	0.225 / 2,000
Number of amino-acid residues	890	890	890
Non-hydrogen atoms	7,853	7,759	7,761
Mean *B* ($Å^2$) protein all/mc/sc	17.9 / 16.1 / 19.6	18.3 / 16.3 / 20.3	25.6 / 23.5 / 27.6
Mean *B* ($Å^2$) solvent / N°. mol.	22.7 / 417	23.3 / 409	27.6 / 411
Rmsd bond lengths (Å), angles (°)	0.008, 1.09	0.008, 1.06	0.007, 1.08
Ramachandran[5]: favored (%) / allowed (%) / Outliers	98.1 / 100 / 0	97.6 / 100 / 0	98.1 / 99.9 / 1
PDB accession code	4PTV	4PTW	4PTX

[1] The outer shell statistics of the reflections are given in parentheses. Shells were selected as defined in *XDS* (Kabsch, 1993) by the user.

[2] $R_{sym} = [\ \Sigma_{hkl} \Sigma_i |I - <I>| / \Sigma_{hkl} \Sigma_i |I|\ ]$

[3] CC(1/2) = Percentage of correlation between intensities from random half-datasets. Values given represent correlations significant at the 0.1% level (Karplus and Diederichs 2012). Shells with CC(1/2) exceeding 50% have been included.

[4] $R_{factor} = \Sigma_{hkl} |\ |F_o| - |F_c|\ | / \Sigma_{hkl} |F_o|$

[5] As determined by *MolProbity* (Lovell et al., 2003).

Table S2. Details of protein-sugar interactions in different *Ho*BGLA complexes

Complex	*Ho*BGLA<br>TCB		*Ho*BGLA<br>*2F*Glc		*Ho*BGLA<br>Glc	
Sugar-protein interactions in subsite –1	O1’	E354 Oε1<br>E354 Oε2<br>N165 Nδ2	C1	E354 Oε1 (cov)	O1	E166 Oε1<br>E166 Oε2<br>H_2O
	O2’	E354 Oε2<br>Q20 Oε1	F2	E354 Oε2<br>N165 Nδ2	O2	E354 Oε2<br>N165 Nδ2
	O3’	H121 Nε2<br>Q20 Nε2<br>W409 Nε1	O3	H121 Nε2<br>Q20 Oε1<br>W409 Nε1	O3	H121 Nε2<br>Q20 Oε1<br>W409 Nε1
	S4’	sulfur linkage, none	O4	Q20 Nε2<br>E408 Oε1	O4	Q20 Nε2<br>E408 Oε1
	O5’	E166 Oε1<br>E166 Oε2<br>E354 Oε1	O5	H_2O	O5	None
	O6’	E166 Oε2	O6	E408 Oε2	O6	E408 Oε2
Sugar-protein interactions in subsite +1	O2	E408 Oε1	–	–	–	–
	O3	E408 O<br>H_2O	–	–	–	–
	O4	3 H_2O	–	–	–	–
	O5	H_2O	–	–	–	–
	O6	None	–	–	–	–

Table S3. Details of protein-sugar interactions for the 3GALA and 6GALA models

Sugar-protein interactions	3GALA		6GALA	
Subsite +1 (R end)	$O1_1$	–	$O1_1$	–
	$O2_1$	–	$O2_1$	Thr224 Og1
	$O3_1$	–	$O3_1$	
	$O5_1$	–	$O5_1$	–
	$O6_1$	Ser297 Og1<br>Ser297 N	$O6_1$	–
	pyranose ring	Trp327 ring	pyranose ring	–
Subsite +1	$O1_2$ (1→4 link)	Asn222 Od1	$O1_2$ (1→4 link)	–
	$O2_2$	Asn222 Od1	$O2_2$	Glu173 Oe1
	$O3_2$	Corresponds to linking oxygen $O1_3$	$O3_2$	His180 Ne2<br>Glu173 Oe1
	$O4_2$	–	$O4_2$	–
	$O5_2$	–	$O5_2$	–
	$O6_2$	Glu408 Oe2	$O6_2$	Corresponds to linking oxygen $O1_3$
	pyranose ring	–	pyranose ring	–
Subsite –1 (NR end)	$O1_3$ (1→3 link)	Glu166 Oe2	$O1_3$ (1→6 link)	Glu166 Oe2
	$O2_3$	Glu354 Oe2<br>Asn165 Nd2	$O2_3$	Glu354 Oe2<br>Asn165 Nd2
	$O3_3$	His121 Ne2<br>Gln20 Oe1<br>Trp409 Ne1	$O3_3$	His121 Ne2<br>Gln20 Oe1<br>Trp409 Ne1
	$O4_3$	Trp409 Ne1	$O4_3$	Trp409 Ne1
	$O5_3$	–	$O5_3$	–
	$O6_3$	Glu408 Oe1<br>Trp401 Ne1	$O6_3$	Glu408 Oe1<br>Trp401 Ne1
	pyranose ring	Trp401 ring	pyranose ring	Trp401 ring

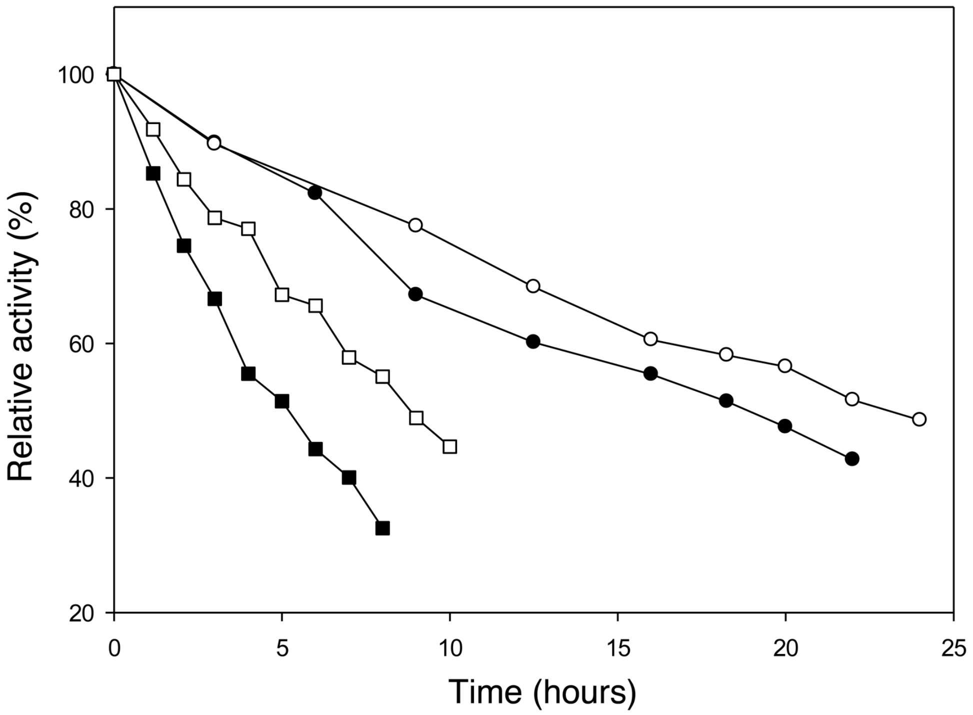

Figure S1. Thermostability of β-galactosidase activity

The thermostability of *Ho*BGLA at 65 and 70°C in 20 mM HEPES and 150 mM NaCl (pH 7.0). *o*NPGal was used as substrate for the enzyme assay. Symbols: (●) without Mg^{2+} and (○) with 1 mM Mg^{2+} at 65°C; (■) without Mg^{2+} and (□) with 1 mM Mg^{2+} at 70°C.

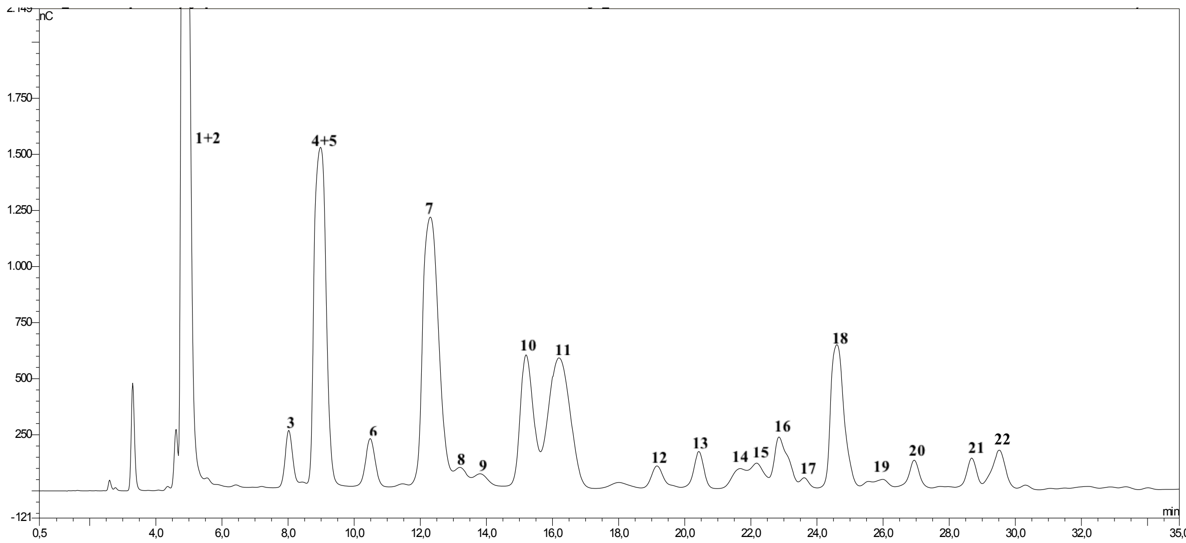

Figure S2. Separation by HPAEC-PAD of individual GOS produced during lactose conversion catalyzed by *Ho*BGLA. The identified compounds are: (1) D-galactose; (2) D-glucose; (3) D-Galp-(1→6)-D-Gal; (4) D-Galp-(1→6)-D-Glc (allolactose); (5) D-Galp-(1→4)-D-Glc (lactose); (6) D-Galp-(1→3)-D-Gal; (7) D-Galp-(1→6)-Lac; (10) D-Galp-(1→3)-D-Glc; (16) D-Galp-(1→4)-Lac; and (18) D-Galp-(1→3)-Lac. Peaks 8, 9, 11-15, 17 and 19-22 were not identified.

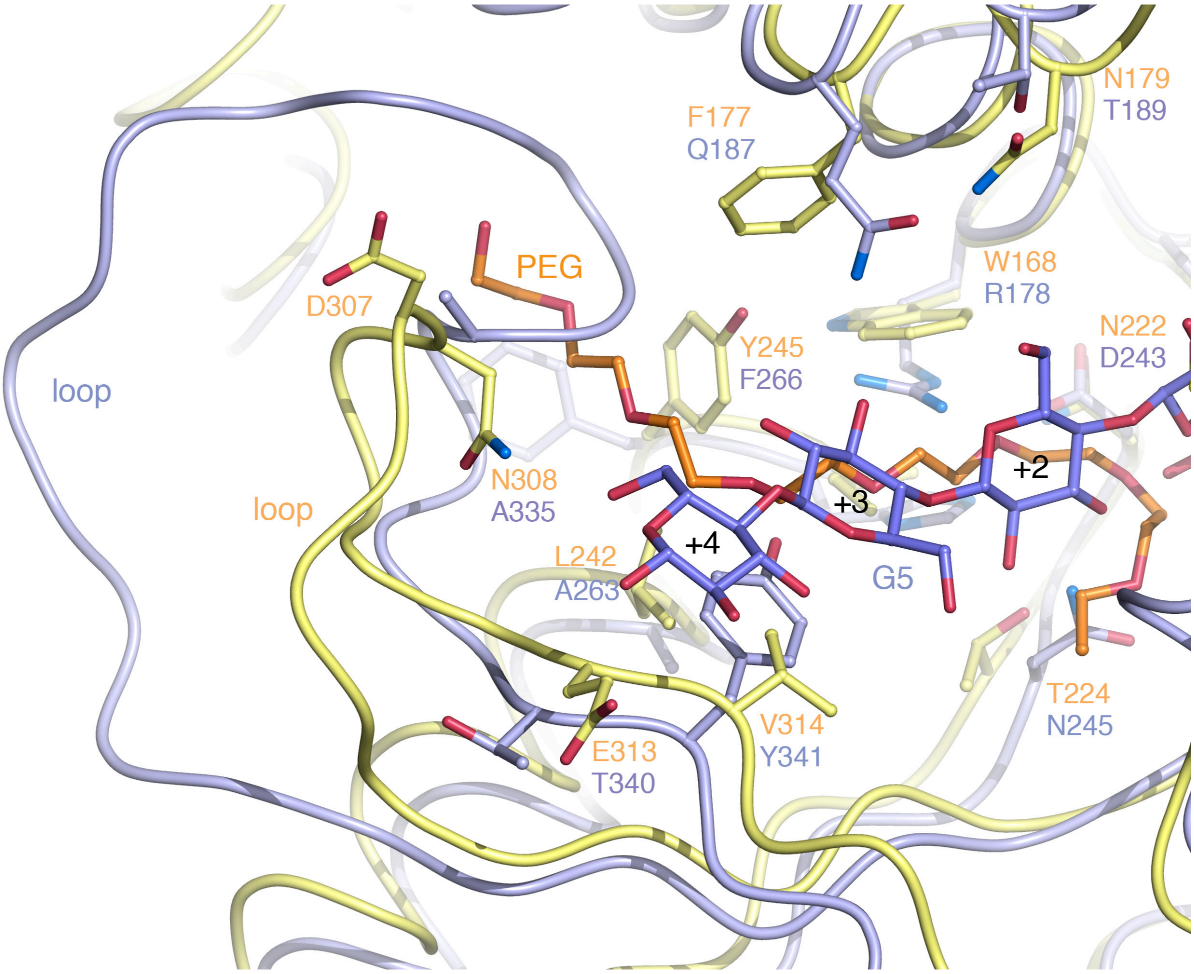

Figure S3. PEG binding by *Ho*BGLA and comparison with cellopentaose binding in BGlu1. Binding of PEG (orange) to *Ho*BGLA (yellow) in subsites +2 to +4 of the substrate-binding cleft. For comparison, the cellopentaose complex (blue) of rice BGlu1 (PDB code 3F5K) has been superimposed. The reducing-end glucosyl of cellopentaose is bound in subsute +4. Numbering in orange refers to that of *Ho*BGLA, and blue for BGlu1. Residues 333–335 of the extended loop in BGlu1 closes off the region where the PEG molecule is bound in *Ho*BGLA.

References

Isorna P, Polaina J, Latorre-Garcia L, Canada FJ, Gonzales B, Sanz-Aparicio J (2007) Crystal structures of *Paenibacillus polymyxa* β-glucosidase B complexes reveal the molecular basis of substrate specificity and give new insights into the catalytic machinery of family 1 glycosidases. J Mol Biol 371:1204–1218.

Kabsch W (1993) Automatic processing of rotation diffraction data from crystals of initially unknown symmetry and cell constants. J Appl Crystallogr 26:795–800.

Karplus PA, Diederichs K (2012) Linking crystallographic model and data quality. Science 336:1030–1033.

Lovell SC, Davis IW, Arendall III WB, de Bakker PIW, Word JM, Prisant MG, Richardson JS, Richardson DC (2003) Structure validation by Cα geometry: ϕ, ψ and Cβ deviation. Prot Struct Funct Genet 50:437–450.

Sansenya S, Opassiri R, Kuaprasert B, Chen CJ, Cairns KJR (2011) The crystal structure of rice (*Oryza sativa* L.) OS4BGlu12, an oligosaccharide and tuberonic acid glucoside-hydrolyzing β-glucosidase with significant thioglucohydrolase activity. Arch Biochem Biophys 510:62–72.

Sue M, Nakamura C, Miyamoto T, Yajima S (2011) Active-site architecture of benzoxazinone-glucoside β-D-glucosidases in Triticeae. Plant Sci 180:268–275.
